# Obesity regulates miR‐467/HoxA10 axis on osteogenic differentiation and fracture healing by BMSC‐derived exosome LncRNA H19

**DOI:** 10.1111/jcmm.16273

**Published:** 2021-01-20

**Authors:** Yijun Wang, Wentao Chen, Liang Zhao, Yadong Li, Zhenyu Liu, Hua Gao, Xiaodong Bai, Baojun Wang

**Affiliations:** ^1^ Departmen of Orthopedics Beijing Friendship Hospital Capital Medical University Beijing China

**Keywords:** bone marrow mesenchymal stem cells, exosomes, fracture healing, obesity

## Abstract

This study explored the therapeutic effect of bone marrow mesenchymal stem cell‐derived exosomes on the treatment of obesity‐induced fracture healing. Quantitative real‐time PCR was used to detect the expression of lncRNA H19, miR‐467 and Hoxa10 and combined with WB detection to detect osteogenic markers (RUNX2, OPN, OCN). Determine whether exosomes have entered BMSCs by immunofluorescence staining. Alkaline phosphatase (ALP) and alizarin red staining (ARS) staining were used to detect ALP activity and calcium deposition. We found that high‐fat treatment can inhibit the secretion of BMSCs‐derived exosomes and affect the expression of H19 carried by them. In vivo and in vitro experiments show that high‐fat or obesity factors can inhibit the expression of osteogenic markers and reduce the staining activity of ALP and ARS. The treatment of exosomes from normal sources can reverse the phenomenon of osteogenic differentiation and abnormal fracture healing. Further bioinformatics analysis found that miR‐467 as a regulatory molecule of lncRNA H19 and Hoxa10, and we verified the targeting relationship of the three through dual luciferase report experiments. Further, we found similar phenomena in ALP and ARS staining. Bone marrow mesenchymal stem cell‐derived exosomes improve fracture healing caused by obesity.

## 1 INTRODUCTION

Fracture is one of the most common traumatic diseases in clinic. Its high incidence, long healing cycle and difficult treatment often bring heavy economic burden to patients.^1^ Although there have been significant improvements in fracture treatment in the past few decades, impaired or delayed fracture healing is still a serious unresolved clinical problem that affects millions of people worldwide.^2^ Studies have shown that poor fracture union/delayed union may be caused by a variety of factors, including comorbidities, among which factors such as obesity, type 2 diabetes (T2D) and other related inflammatory reactions are important risk factors.^3^, ^4^ With the rapid development of society and economy, the changes in people's dietary habits and the lack of exercise in human life and the imbalance of diet and nutrition, the incidence of obesity has shown a trend of increasing year by year.^5^ In the next few decades, this number will continue to increase greatly. Therefore, in the future treatment, poor fracture healing/delayed healing caused by factors such as obesity or high fat will become one of the factors that cannot be ignored in clinically affecting fracture healing.

In *vivo* experiments show that the fracture healing speed of obese rats is slower than that of normal rats, and obesity may be an important reason for the slowdown of fracture healing.^6^ This part of the results has been confirmed in clinical case‐control studies.^4^ In the process of fracture healing, normal physiological inflammation usually plays a positive role. Most normal physiological inflammation is beneficial to the body and is an important defence response of the body. However, the chronic and persistent inflammatory response state caused by obesity will inhibit the differentiation of osteoblasts and stimulate the activation of osteoclasts, destroying the relative balance between the two in the fracture healing process, thereby affecting the normal fracture healing.^7^ Regarding the cause of poor fracture healing caused by obesity, some scholars believe that the lipid content in the plasma of obese patients is higher than normal, and the abnormal lipid metabolism further leads to the restriction of the bone formation process of fracture patients. However, why does an increase in blood lipids lead to a decrease in osteoblast differentiation? How does it work?

Bone marrow mesenchymal stem cells (BMSCs) belong to the self‐renewing pluripotent cell group, which can differentiate into osteoblast cell lines and other cell lines in response to the stimulation of various environmental factors and can be used in tissue regeneration and fracture play a key role in healing.^6^ BMSCs have osteogenic potential, and it can self‐renew and differentiate into mesenchymal‐derived tissues, making it an important contributor to the bone repair process.^7^ In addition, it is reported that the osteogenesis of bone marrow mesenchymal stem cells promotes the regeneration of the skull.^8^ In recent years, as a new means of intercellular communication, exosomes (exo) have attracted much attention because of their ability to carry a variety of biologically active molecules that regulate the activity of receptor cells.^9^ Exosomes are small extracellular vesicles with a diameter of 40‐150nm that can be secreted by a variety of cells. They are present in almost all body fluids, including blood, cerebrospinal fluid (CSF), urine, saliva and breast milk.^10^ The amount of secretion of exosomes and the content of their contents vary with their biogenesis, cell source and extracellular environment. Studies have shown that exosomes may carry a large amount of DNA, mRNA, protein and non‐coding RNAs (ncRNA).^11^ Since exosomes can stably carry certain biological effect molecules and specific proteins, the discussion of the mechanism of the influence of exo functions and contents provides a basis for the development of targeted treatments of diseases and exogenous molecular interventions.

Long non‐coding RNAs (lncRNAs) are a type of RNA with a length of more than 200 nt, and do not participate in the function of encoding proteins (not including rRNA), and are widely distributed in various organisms. lncRNAs participate in cell epigenetics, transcription and post‐transcriptional regulation processes at multiple levels and play an important role in the activities of life organisms.^9^, ^10^ Recent studies have shown that lncRNA is involved in X chromosome silencing, genome imprinting and chromatin modification, transcription activation, transcription interference, nuclear transport and other important regulatory processes. These regulatory effects of lncRNAs have also begun to attract widespread attention.^11^ Recent studies have shown that some lncRNAs are involved in regulating the adipose differentiation and osteogenic differentiation of BMSCs.^12^, ^13^ Studies have reported that LncRNA H19 can stimulate the osteogenic differentiation of BMSCs, while inhibiting the adipogenic differentiation of BMSCs.^14^, ^15^ Our previous research results indicate that LncRNA H19 adsorbs miR‐188 through the ceRNA mechanism and then mediates LCoR’s influence on the osteogenic and adipogenic differentiation of mouse BMSCs,^15^ but does LncRNA H19 pass externally? Exosome carrying and its specific mechanism of action in osteoblast differentiation is not fully understood. In addition, whether H19 is involved in the regulation of obesity fracture healing process has not been reported yet.

In this study, we observed the imaging, histological and molecular changes in the fracture healing process of obese mouse models induced by high‐energy diet to understand the effect of obesity on fracture healing; at the same time, the mouse BMSCs and their exosomes were isolated and cultured. To detect the expression level of H19 in the serum samples of fracture patients and BMSC samples of the above mouse models and then to explore the effect of osteogenic differentiation in a high‐fat environment and the H19 and downstream signals in BMSC‐derived exosomes during this process, the function and mechanism of the pathway will further strengthen our understanding of the mechanisms related to the clinical manifestations of obese fracture patients and provide new research targets and treatment approaches for improving the prognosis of such patients.

## 2 MATERIAL AND METHODS

### 2.1 Clinical sample

Collect plasma samples from 20 healthy fracture patients and 20 obese fracture patients from Beijing Friendship Hospital. Prior to the study, all participants had signed an informed consent form. The criteria for obese fracture patients to enter this study are that the body mass index (BMI) is greater than 30 kg/m^2^, and there is no major disease or pregnancy. Healthy fracture patients were 34‐67 years old, weighed 65‐138 kg and had a body mass index of 18.5‐25 kg/m^2^. The clinical sample information is shown in Table S1. This study was approved by the Ethics Committee of Beijing Friendship Hospital.

### 2.2 Patient plasma preparation and exosomes isolation

Whole blood was collected from patients in the control group and obese group and then centrifuged. Collect the plasma from the test tube and place it in aliquots of 2‐mL cryotubes. Then, 2 mL of the whole blood was put into 50‐mL polystyrene tubes, diluted 1:2 with RPMI (BioWhittaker, cat. no. 12‐167Q), underlaid with 10 mL of Ficoll‐Paque Plus (GE Healthcare, cat. no 17‐1440‐03). Then, centrifuge the tube at 1600 rpm at room temperature for 20 minutes. The white blood cell layer (including lymphocytes and monocytes) on top of Ficoll was transferred to a new 50‐mL test tube. Exosomes were prepared from remaining cell‐free plasma by differential ultracentrifugation with filtration steps.^16^

### 2.3 Animals

Male C57BL/6J mice at 3‐week age were purchased from The Changsheng company (Liao‐ning, China) and allowed to acclimate to the care facility for 1 week. Mice were group‐housed in ventilated cages with bedding and provided with ad libitum access to pelleted feed and water. The mouse facility had a 12‐hour light/dark cycle at 21.1°C to 22.8°C and 30% to 70% humidity.

### 2.4 Induction of obesity and fracture

Three‐week‐old SPF‐grade C57BL/6J male mice were randomly divided into two groups, the model group and the control group, and were raised in IVC boxes with 4 mice per box. From 7 weeks of age, the model group was fed high‐fat diet and the control group was fed normal diet. Observation method: During the obesity model, the weight and length of the mice were measured every week, and Lee's index was calculated and recorded.

Fracture building before 6 hours to ban the eating and drinking water in mice, measuring weight, use the configured by intraperitoneal injection of 10% chloral hydrate in anaesthesia, rate of 1 mL/100 g dose injection, after being completely anaesthesia in mice, the skin right lower limb, disinfection, mice, the mice on the right side of the knee joint, a longitudinal incision under cut 0.5 cm, the control group using 0.9% saline flushing after stitching it step by step. In the experimental group, 22G/0.41 mm intramedullary fixation needles were first inserted from the kneecaphim, and the muscles and fascia were separated at the middle and upper 1/3 of the tibia. After the tibial diaphysis was sawn off with a saw blade, 0.9% normal saline was immediately used to wash the tibial surface, and then, the intramedullary fixation needles were completely inserted and aligned. A mouse model of right tibial shaft fracture was established by sutured incisions layer by layer. After operation, the environment was cleaned and single cage was raised at room temperature of 22‐24°C, humidity of 60%, regular ULTRAVIOLET disinfection and ventilation. The mice were allowed to move freely and were fed the corresponding diet according to the group of previously obese and normal mice.

The mice were then randomly assigned to four groups: Control, HFD, HFD‐PBS or HFD‐Exo (n = 8/group). Next, HFD‐Exo (200 μg of total protein of exosomes precipitated in 200 μL of PBS) or an equal volume of PBS (200 μL) was injected immediately near the fracture followed by wound closure and suture.

### 2.5 Cell culture

The BMSCs were extracted and passaged according to the previous operation methods of our research group.^15^ The P3 of BMSC was digested and passaged, counted on a counting plate and seeded in a 6‐well plate at a cell density of 5 × 10^4^/mL. After the cells were approximately 80% fused, the conditioned medium was replaced to start the grouping experiment. The experiment was divided into a control group and a high‐fat group. The control group was added with traditional osteoinductive agents (dexamethasone 10‐8 mol/L, β‐glycerophosphate sodium 10 mmol/L, vitamin C 50 mg/L α‐MEM complete medium); high‐fat group was added with high‐fat osteogenic induction solution (ie the α‐MEM complete medium in the traditional osteogenic induction solution is changed to a high‐fat medium). Transform BMSC into osteoblasts, change the culture medium once every 3 days, observe the cell growth morphology and take pictures under an inverted phase contrast microscope.

### 2.6 Isolation and identification of exosomes from BMSCs

When the BMSCs reached 80% confluence, the medium was replaced with exosome‐free medium, and the supernatant was collected 48 hours later. Subsequently, differential centrifugation and filtration were performed to separate exosomes from the supernatant of BMSC. The cell supernatant was centrifuged at 2000 g for 20 minutes, then at 10 000 g for 40 minutes and then filtered through a 0.22‐μm sterile filter. The supernatant was then ultracentrifuged at 100 000 g for 70 minutes, resuspended in PBS, centrifuged at 100 000 g for 70 minutes to remove any remaining RNA and diluted with a mixture containing PBS and RNAse I.

The morphology of exosomes was observed under transmission electron microscope (TEM). Images were obtained with HT7700 transmission electron microscope (Hitachi, Japan) at 120kV. The particle size and concentration of exosomes were determined by nanoparticle tracking analysis (NTA). Western blotting was used to detect exosome‐specific markers.

### 2.7 Osteogenic differentiation

After 7 days incubation, cells were harvested artificially and tested ALP activity with an Alkaline Phosphatase Assay kit (Abcam) the procedures proceeded in accordance with manufacturer's instructions. ALP activity was recorded at 405 nm using an ALP activity assay kit (Jiancheng) as previously described.^17^ Total protein content of each sample was determined with a BCA kit (Beyotime). ALP activity relative to the control group was normalized to the total protein content After 14 days, and Alizarin Red staining (ARS) was performed. ARS was quantified as described.^18^ The samples placed on a Flex Station 3 microplate reader (Molecular Devices) at 405 nm in 96‐well opaque‐walled transparent‐bottomed plates. For semi‐quantitative evaluation of the degree of mineralization, 100 mmol/L cetylpyridinium chloride (Amresco, America) was employed to elute the stain for 30 min and quantified by spectrophotometric absorbance at 570 nm.

### 2.8 Quantitative real‐time PCR (QRT‐PCR)

Fracture callus tissues were collected after fracture. Primers were designed by using the Primer 5.0 Express software. The sequences were shown in Table S2. Briefly, a commercial kit (Qiagen) was used to purify RNA from fracture callus tissue. Subsequently, purified RNA was undergoing a reverse transcription procedure by adopting SuperScript Ⅲ First Strand Synthesis System (Invitrogen). At last, SYBR Green intercalating dye (Molecular Probes) was used to perform real‐time PCR with the Gene Amp PCR System 9700 (Applied Biosystems, Thermo Fisher Scientific). Values for relative expression were processed using the 2^−ΔΔ^ *^Ct^* approach. GAPDH and U6 was the intern control.

### 2.9 Western blot (WB)

First of all, proteins were isolated from extraction reagent (Thermo Fisher Scientific), followed with separated and transferred some proteins to Immobilon™‐P membranes (Merck Millipore). Second, incubating Hoxa10, runt‐related transcription factor 2 (Runx2), osteopontin (OPN), osteocalcin (OCN) and GAPDH (Abcam) primary antibodies against mouse in the membrane at 4°C for a night time. Washed with TBST after incubation immediately and hybridized membranes with horseradish peroxidase (HRP)‐linked antibody goat anti‐rabbit IgG (1:2000, Abcam) for 1 hour. At least but not last, checking the antibody binding by an enhanced chemiluminescence kit.

### 2.10 Dual luciferase reporter assay

To identify the target of miR‐467, putative target genes were searched using Targetscan software (www.targetscan.org/). Wild‐type or mutated Hoxa10 and H19 3'UTR including the predicted miR‐467 binding site were PCR‐amplified and inserted into the pMIR reporter plasmid. The luciferase reporter plasmids of wild‐type or mutated Hoxa10 and H19 were transfected into cells, respectively, and miR‐467 mimics were also transfected. Luminescence was measured 48 hours after transfection using a dual luciferase detection kit (Promega), according to the manufacturer's instructions. Measurements of luminescence were performed on a GloMax 20/20 Luminometer (Promega Corporation).

### 2.11 Ribonucleoprotein immunoprecipitation assay

Anti‐AGO2 ribonucleoprotein immunoprecipitation (RIP) was performed in HEK‐293T cells transfected with miR‐467 mimics or miRNA. Briefly, HEK‐293T cell lysates were pre‐blocked with Protein G beads (Invitrogen) and then incubated with anti‐AGO G beads (Pierce Biotechnology) at 4°C for 90 minutes. Beads were collected by centrifugation at 600 × *g* for 1 minute, washed 5 times with RIPA buffer and resuspended in Tris‐HCl 50 mmol/L (pH 7.0). The beads were then incubated 45 minutes at 70°C to reverse the crosslinks, and the RNAs that co‐IP with anti‐AGO antibodies were extracted using TRIzol (Invitrogen) following the manufacturer's instructions and then quantified by RT‐PCR.

### 2.12 Immunofluorescence staining

Exosomes were labelled with the red fluorescent cell linker PKH26 according to the manufacturer's instructions. Each treatment group was diluted in 1 mL diluent C, and 4 μL PKH26 dye was diluted in 1 mL diluent C. The dilutions were then mixed gently for 4 minutes, and 2 mL of 0.5% bovine serum albumin (BSA) was added to bind excess dye. The labelled exosomes were washed in PBS at 100 000 g for 70 minutes. BMSCs were then incubated with different concentrations of labelled exosomes for 4 or 24 hours. After incubation, the cells were washed twice with PBS and fixed in 4% paraformaldehyde for 10 minutes. Cellular nuclei were stained with 6‐diamidino‐2‐phenylindole (DAPI) solution at 1 μg/mL. The exosome uptake images were captured with an LSM 5 EXCITER confocal laser scanning microscope (Carl Zeiss).

### 2.13 Bone mineral density detection of fracture areas

After removal of the internal fixatives, the femora were subjected to detect bone mineral density (BMD) of the 2.0 mm × 1.5 mm rectangular region centred on the fracture by a dual‐energy X‐ray absorptiometry (Lunar DPX‐NT, General Electric Company) using fast scanning mode.

### 2.14 Bone mechanical testing

The mechanical properties of the femora were determined 6 weeks after fracture using a 3‐point bending test. Before the test, frozen femora were thawed at 4 ˚C overnight. A material test machine (H23Ks, Hounsfield Test Equipment, UK) with a 10 N load cell was used. The femora were positioned horizontally in a fixture attached to a testing machine with their anterior surface upwards, centred on the supported. Load was applied vertically and onto the fracture site at the rate of 5 mm/min. The load versus displacement curve was recorded, and ultimate load, stiffness and the energy absorbed to failure values were calculated by built‐in software (QMAT Professional; Tinius Olsen). Contralateral intact femora were also evaluated for the calculation of relative mechanical properties.

### 2.15 Statistical analysis

GraphPad Prism 6.0 software (GraphPad Software) was used for statistical analysis, and data were expressed with mean ± standard deviation. Data were compared by unpaired *t* test (differences between two groups) or one‐way ANOVA analysis (differences among groups). *P value < .05 was regarded statistically significant.

## 3 RESULT

### 3.1 Characterization of exosomes derived from BMSCs

**FIGURE 1:**
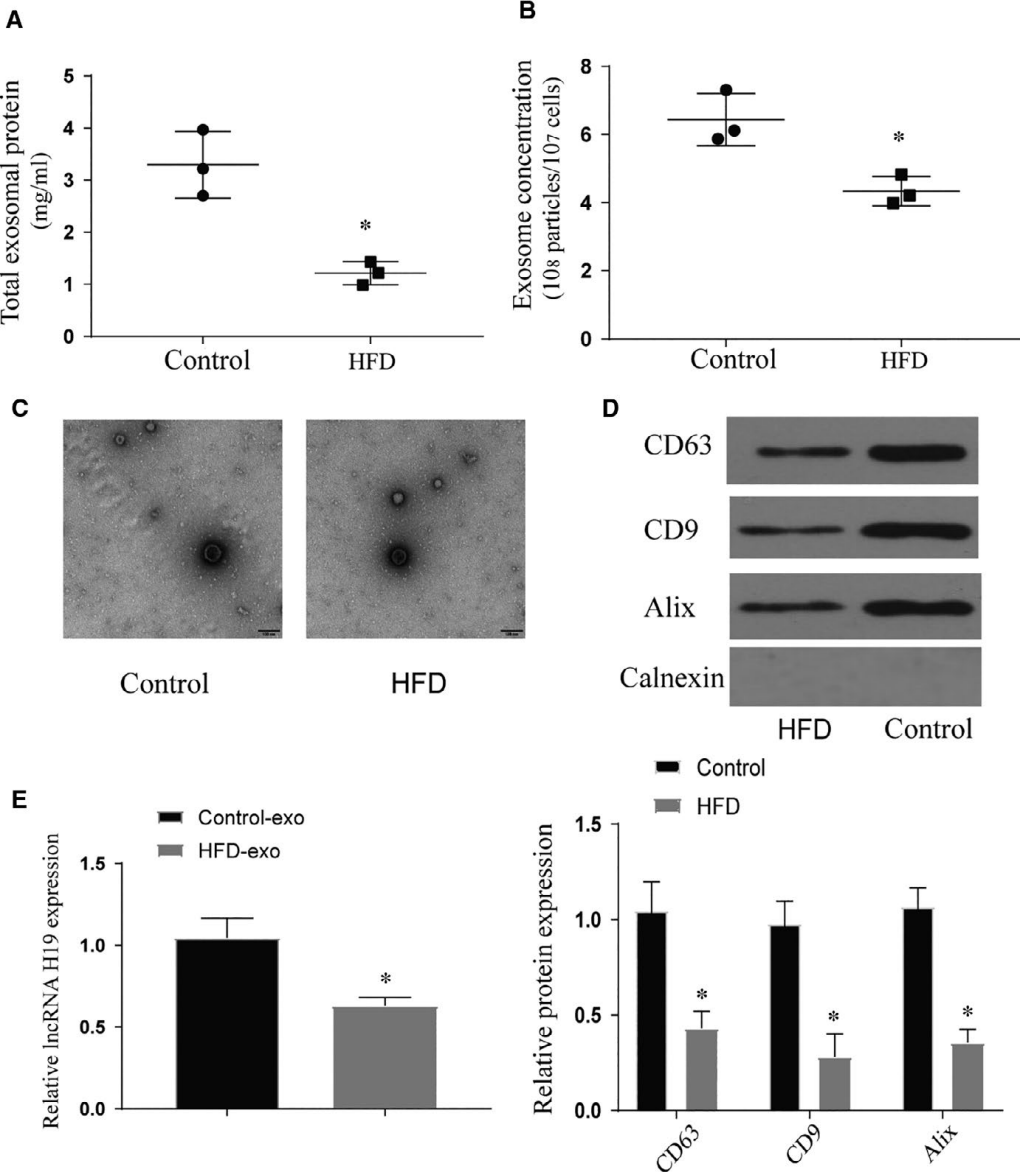
Characterization of exosomes derived from BMSCs. (A) Total exosomal protein expression, *vs control *P* < .05; (B) The concentration of exosomes secreted in each treatment group, *vs control *P* < .05; (C) Morphology of exosomes in each treatment group under electron microscope; (D) Expression of exosomal markers in each treatment group; (E) The expression of lncRNA H19 carried by exosomes in each treatment group, *vs control *P* < .05. All data were means ± SD

With reference to previous research methods,^19^ we tested the total protein of exosomes derived from BMSCs after two different treatments and found that the total protein of exosomes after high‐fat treatment was significantly lower than the control group. Further NTA analysis found that exosomes secretion decreased after high‐fat treatment (*P* < .05) (Figure 1A‐C). As the exosomes were isolated from equivalent numbers of cells, the intensity of the exo markers reflected the ability of cells to secrete exos. High‐fat treatment led to a significant decrease of Alix, CD63 and CD9 in the ultracentrifuged pellets but not in the control, suggesting that the differences in secretion levels are due to impaired secretion of exos (*P* < .05) (Figure 1D). After PCR detection, it was found that the content of H19 carried by exosomes was also significantly lower than the control group (*P* < .05) (Figure 1E). These results indicate that the high‐fat treatment leads to a decrease in the secretion of BMSCs‐derived exosomes and a significant decrease in the H19 content carried by them.

### 3.2 Effects of obesity and high‐fat factors on osteogenic differentiation of BMSCs

**FIGURE 2:**
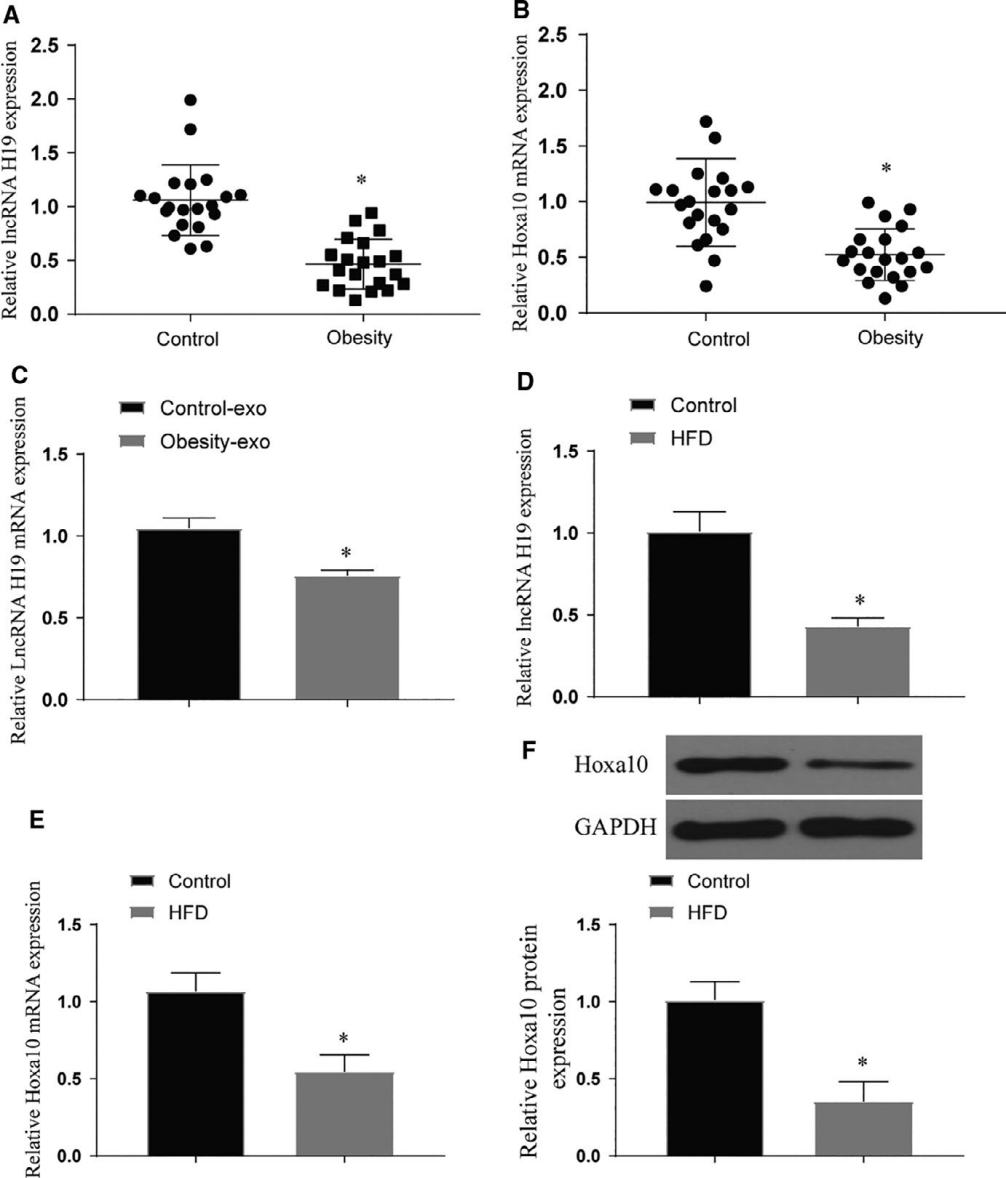
Expression of lncRNA H19 and Hoxa10 in clinical samples and BMSCs after high‐fat treatment. (A‐B) Expression of lncRNA H19 and Hoxa10 in clinical samples, *vs control *P* < .05; (C) Expression of lncRNA H19 and Hoxa10 in exosomes of clinical samples, *vs control *P* < .05; (D‐F) Expression of lncRNA H19 and Hoxa10 after high‐fat treatment of BMSCs, *vs control *P* < .05. All data were means ± SD

**FIGURE 3:**
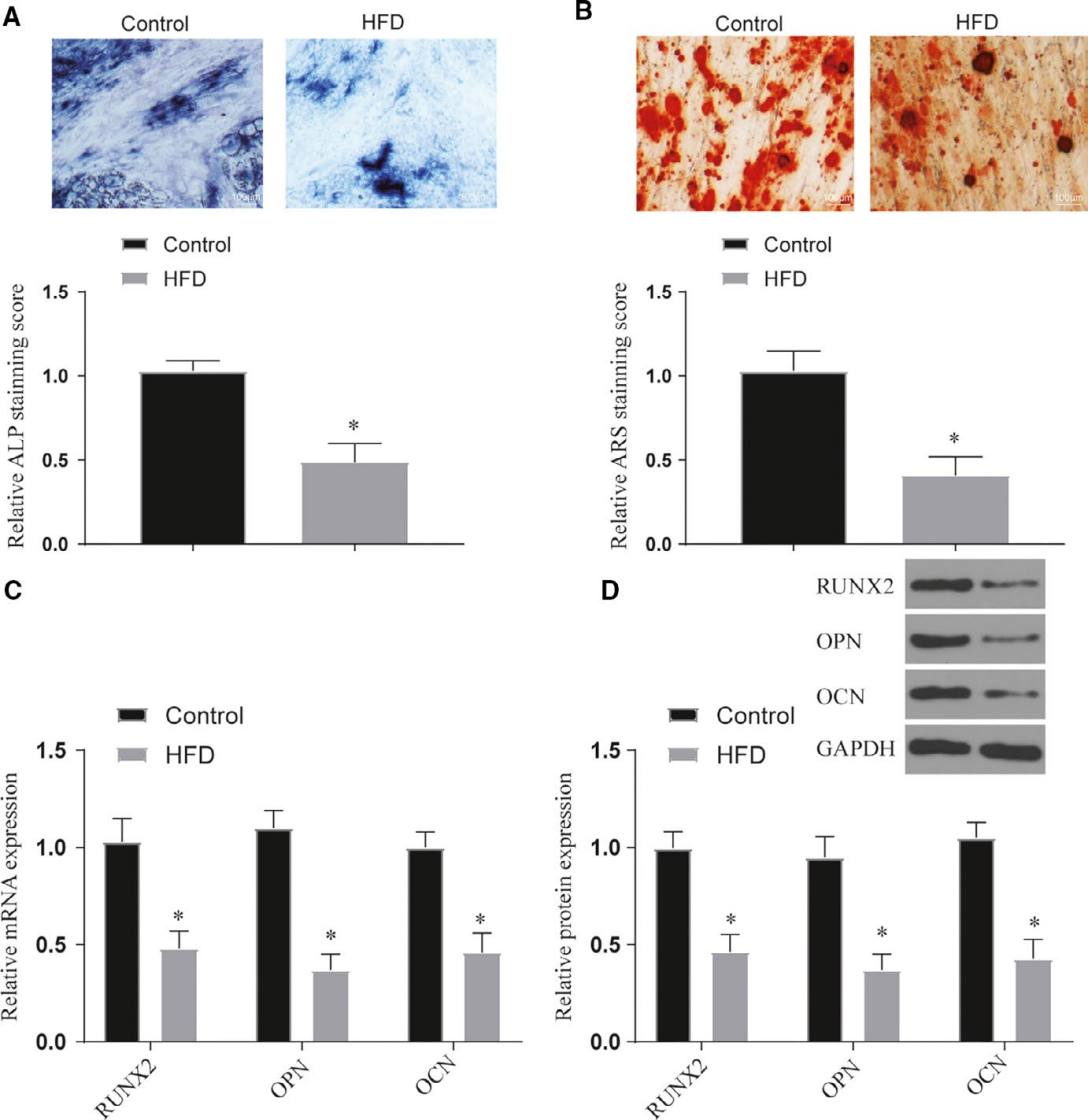
Effects of high‐fat treatment on osteogenic differentiation of BMSCs in vitro. (A‐B) Staining of ALP and ARS in each treatment group after high‐fat treatment, original magnification × 200. *vs control *P* < .05; (C‐D) The expression of bone marker genes and proteins in each treatment after high‐fat treatment, *vs control *P* < .05. All data were means ± SD

**FIGURE 4:**
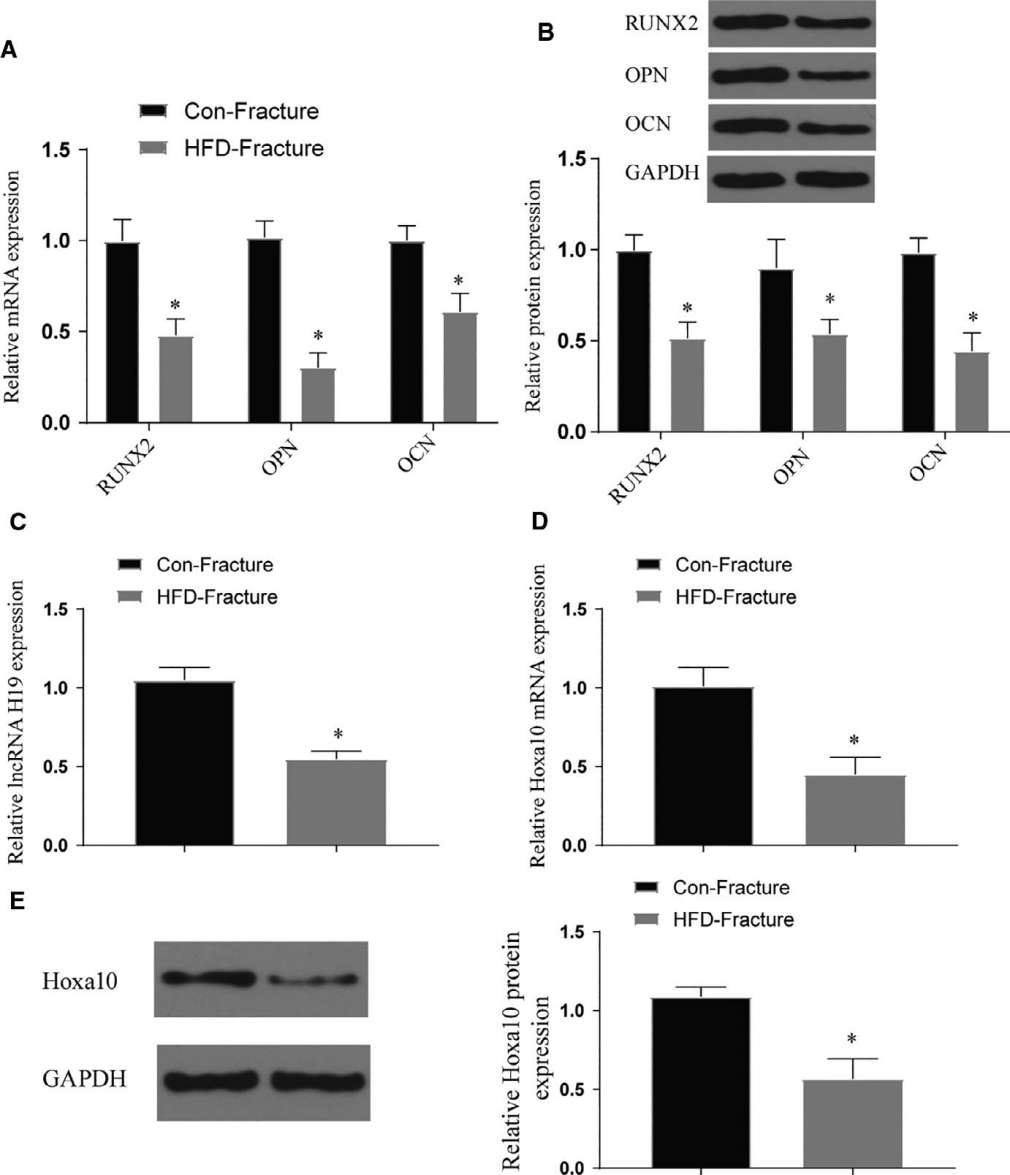
The effect of high‐fat diet on the osteogenic differentiation of fracture models in vivo. (A‐B) The expression of bone marker genes and proteins in tissues at the fracture site after high‐fat treatment, *vs control *P* < .05; (C‐E) Expression of lncRNA H19 and Hoxa10 after high‐fat treatment of mice, *vs control *P* < .05. All data were means ± SD

After analysing the lncRNA of obese and normal fracture patients, we found that the expression of H19 in obese patients was significantly lower than that in the control group. In addition, the expression of Hoxa10 decreased in obese patients (*P* < .05) (Figure 2A,B). At the same time, we extracted exosomes from two sets of blood samples for lncRNA expression detection and the results were the same as above. It proves that obesity factors affect the expression of H19 in exosomes (Figure 2C). In vitro experiments showed that the expression levels of H19 and Hoxa10 of BMSCs treated with high fat were significantly decreased (*P* < .05) (Figure 2D‐F). After staining with ALP and ARS, we found that high‐fat treatment can significantly inhibit the osteogenic differentiation of BMSCs (*P* < .05) (Figure 3A,B). This phenomenon also appears in the detection of marker genes for osteogenic differentiation (*P* < .05) (Figure 3C,D). Further mouse fracture model verification, the fracture group of high‐fat diet also showed the phenomenon of decreased expression of osteogenic marker genes (*P* < .05) (Figure 4). Both results show that high‐fat diet can inhibit osteogenic differentiation, and this inhibition is related to the increased expression of H19 and Hoxa10.

### 3.3 Therapeutic effect of exosomes on osteogenic differentiation and poor fracture healing caused by obesity factors

**FIGURE 5:**
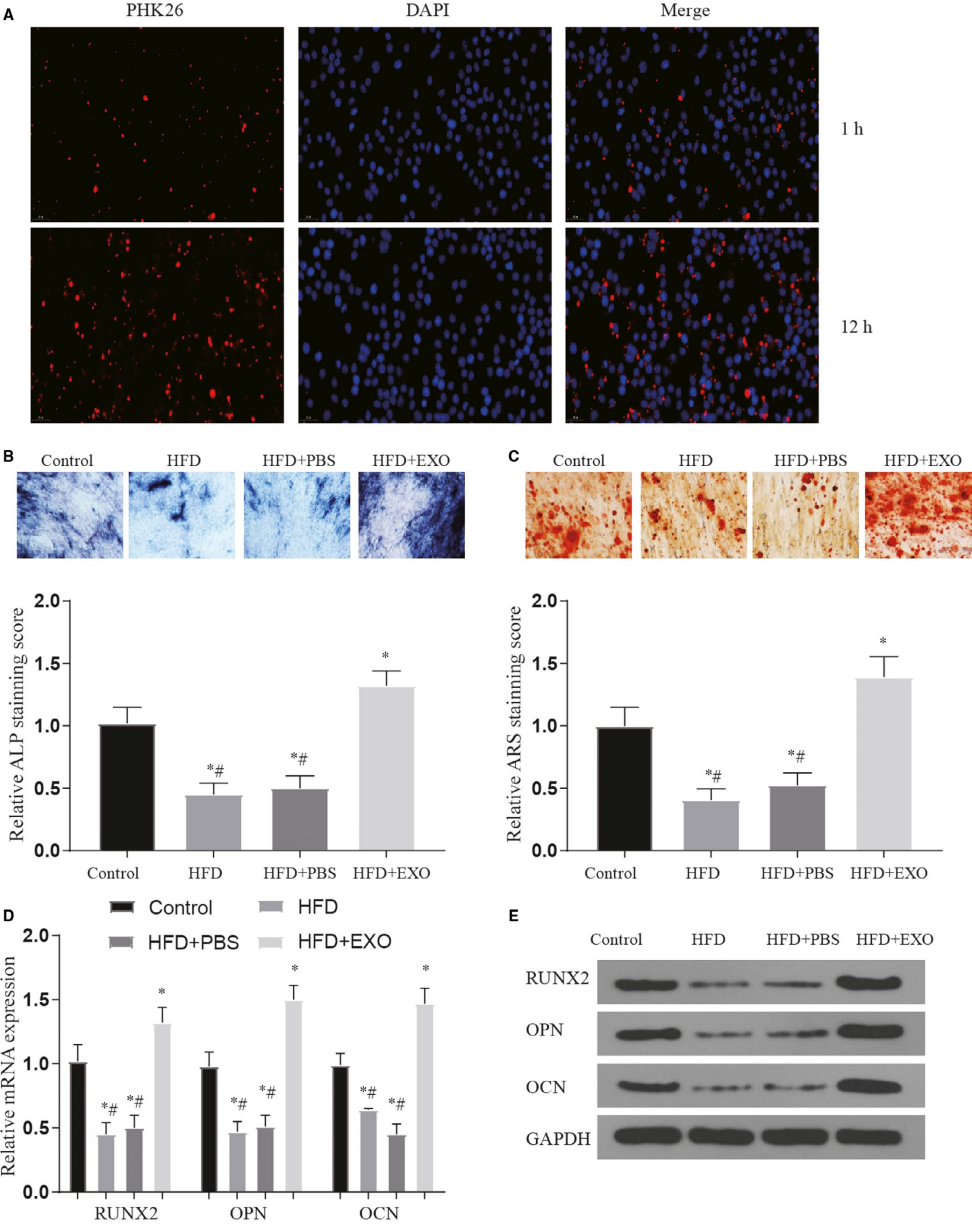
The therapeutic effect of exosomes on abnormal osteogenic differentiation caused by high‐fat treatment. (A) PKH26 labelled exosomes detected exosomes successfully entered BMSCs after processing over time; (B‐C) staining of ALP and ARS in each treatment group after exo treatment, Original magnification × 200. *vs control *P* < .05, #vs HFD + EXO group *P* < .05; (D‐E) the expression of bone marker genes and proteins in each treatment after exo treatment, *vs control *P* < .05, #vs HFD + EXO group *P* < .05. All data were means ± SD

**FIGURE 6:**
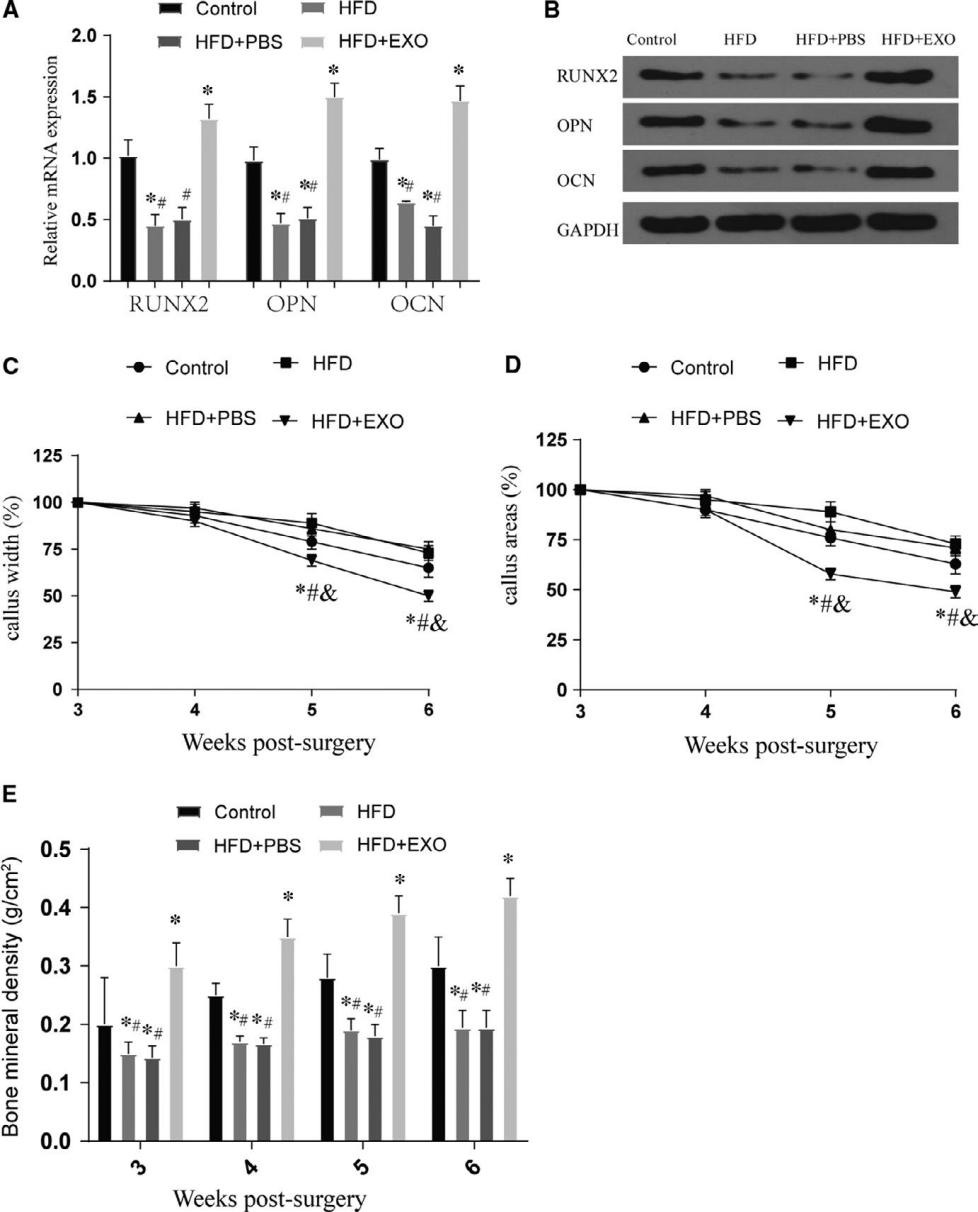
The effect of exosomes on fracture healing. (A‐B) The expression of bone marker genes and proteins in tissues at the fracture site after exo treatment, *vs control *P* < .05, #vs HFD + EXO group *P* < .05; (C‐D) callus width and area are measured on plain X‐ray image and normalized to initial reading at week 3 post‐fracture, *vs control *P* < .05, #vs HFD + EXO group *P* < .05; (E) BMD of fracture area was detected at week 3 to 6 in all groups, *vs control *P* < .05, #vs HFD + EXO group *P* < .05. All data were means ± SD

Through the above test, the reduction of BMSC‐derived exosomes and the reduction of H19 expression after HFD treatment may be the main reason for the poor fracture healing caused by obesity. Therefore, we tried to judge whether this therapeutic effect is real by applying normal processed exosomes. After the exo treatment, we found that compared with the PBS group, the expression of H19 and Hoxa10 increased significantly (*P* < .05) (Figure S1a,b). The detection of PKH26‐labelled exosomes showed that the number of exosomes entering BMSCs increased significantly over time (Figure 5A). Furthermore, we found through the detection of osteogenic differentiation specific staining and osteogenic marker genes that the application of exo treatment can effectively improve the phenomenon of poor osteogenic differentiation caused by high‐fat treatment. These results are similar to our expectations (*P* < .05) (Figure 5B‐E). To further examine the effects of exosomes on bone formation of BMSCs, a series of in *vivo* experiments were performed. Quantitative analysis showed that callus width and area decreased gradually along bone healing, which was the most significant in EXO group. And the decreasing of callus width and area in the HFD group was the slowest. Results of BMD detection showed BMD in fracture areas increased gradually along in all groups. Meanwhile, compared with control group or HFD + PBS group, BMD was significantly higher in the EXO group (*P* < .05) (Figure 6). The results of the 3‐point bending test (Figure S2a,c,e) showed that the maximal loading for failure, stiffness and energy absorbed for failure were higher in HFD + EXO group compared with the other group (*P* < .05). The relative mechanical properties (maximum load, stiffness and energy absorbed for failure) to collateral side were also detected (Figure S2b,d,f). Data normalization to normal lateral femora showed that HFD + EXO group remains effective in enhancing maximum load, stiffness and energy absorbed for failure (*P* < .05). The results of in vivo and in vitro experiments show that exosomes can effectively improve the osteogenic differentiation and abnormal fracture healing caused by obesity.

### 3.4 Verification of the targeting relationship between miR‐467 and lncRNA H19 and Hoxa10

We verified that BMSC‐derived exosomes after different treatments can affect osteogenic differentiation and fracture healing. And this effect is exerted by regulating the expression of lncRNA H19 and Hoxa10. Therefore, we tried to find the intermediate link of their regulation and verify this phenomenon. We use the Starbase database to predict the target miRNA of H19 and Hoxa10. After screening the three miRNAs targeting the same miRNA by means of Venn diagrams, it was found that miR‐467 may play an important role in it (Figure S3a). First, we also verified in clinical samples that miR‐467 is highly expressed in obese patients (Figure S4). Then, we used the Starbase database to predict their possible binding sites, and through the dual luciferase reporter assay and RIP assay, we proved that the three are mutually binding (Figure S3b‐e). Furthermore, after verifying the downstream target genes by overexpression of H19 and miR‐467, we found that LncRNA can competitively bind miR‐467 to affect the expression of Hoxa10 (*P* < .05) (Figure S3f‐g).

### 3.5 The role of miR‐467 in lncRNA H19 in regulating osteogenic differentiation

**FIGURE 7:**
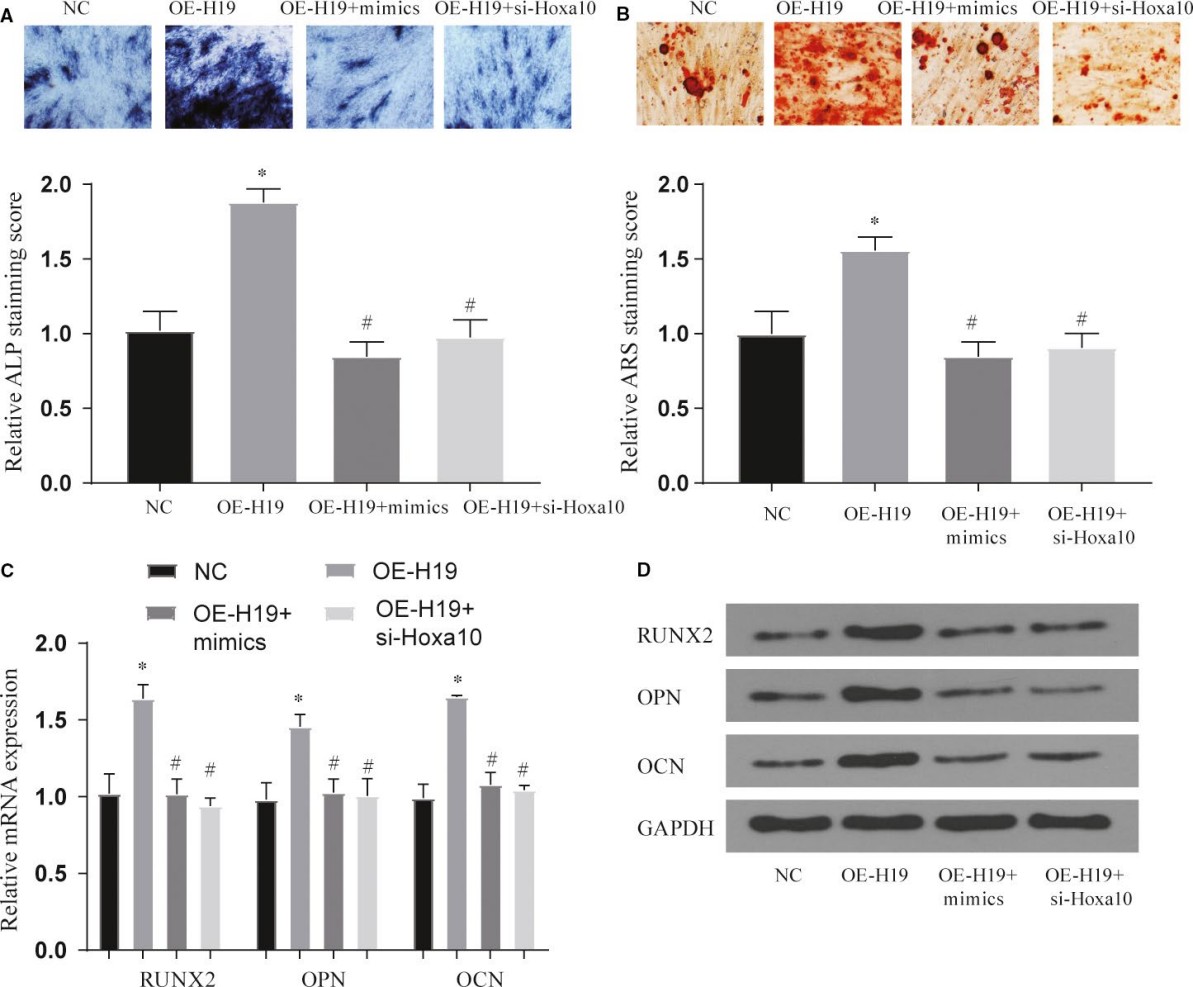
Effects of treatment groups on osteogenic differentiation of BMSCs. (A‐B) Effect of each treatment group on ALP and ARS staining, original magnification × 200. *vs NC group *P* < .05, #vs OE‐H19 group *P* < .05; (C‐D) Effect of each treatment group on bone marker genes and proteins, *vs NC group *P* < .05, #vs OE‐H19 group *P* < .05. All data were means ± SD

First, we verified the success of our gene interference model (Figure S1c‐e). Similarly, we verified by staining with ALP and ARS that the overexpression of miR‐467 can reverse the phenomenon of promoting osteogenic differentiation produced by H19 (*P* < .05) (Figure 7A‐B). And this reversal effect also appeared when Hoxa10 was inhibited. Furthermore, we detected the osteogenic marker genes through qPCR and WB experiments and found that the results were similar to the staining results (*P* < .05) (Figure 7C‐D). These experiments verified that lncRNA H19 competitively binds to miR‐467 by regulating the expression of Hoxa10, thereby affecting osteogenic differentiation.

## 4 DISCUSSION

With the deepening of research on exosomes, in addition to normal cell‐to‐cell communication, they can be well used in targeted treatment of diseases.^20^ In this study, we first verified that obesity or high‐fat environment can affect the secretion of exosomes derived from BMSCs and abnormal osteogenic differentiation. This research group and a large number of previous studies have confirmed that H19 can affect osteogenic differentiation, but its upstream sources and participating mechanisms have not been discussed in depth.^15^, ^17^, ^21^ We have confirmed for the first time that the reduction of H19 expression is caused by the reduction of exosomes secreted and carried by a high‐fat environment. We have proved through in vivo and in vitro experiments that normally processed BMSCs‐derived exosomes can increase the expression of H19 and play a role in the treatment of osteogenic differentiation and poor fracture healing caused by obesity. Further through bioinformatics methods, we verified that H19 can affect Hoxa10 expression by competitively binding miR‐467, thereby affecting osteogenic differentiation. These studies can provide new treatment ideas for the treatment of poor fracture healing caused by obesity.

In clinical practice, we often find that the healing time and healing degree of the fracture of obese fracture patients are weaker than those of normal weight fracture patients. This interesting phenomenon has attracted the attention of this research group. Reading previous research literature found that there have been reports verifying abnormal osteogenic differentiation or poor fracture healing caused by obesity.^22^, ^23^ In this study, we also verified this phenomenon through in vivo and in vitro experiments. Exosomes have been proven to affect cell functions as an important link in cell‐to‐cell communication.^20^ The previous research of our group has confirmed that H19 can affect the osteogenic differentiation of BMSCs. In this study, after constructing high‐fat‐treated BMSCs, it was found that the secretion of exosomes was significantly reduced. After further testing of the H19 carried by the BMSCs, it was found that the high‐fat treatment can also affect the expression of H19. These results indicate that obesity factors may affect the osteogenic differentiation and fracture healing by affecting the exosomes derived from BMSCs to carry H19.

The molecular determinants of positional identity in regenerating bone remain subject to substantial debate but a cardinal role of Homeobox (HOX) genes, which provide spatial information affecting both bone position and whole‐body morphology in vertebrates, in determining positional identity in this respect has remained unchallenged. HOX genes constitute a family of evolutionarily‐conserved transcription factors that regulate patterning of the developing skeleton.^24^ Studies have shown that Hoxa10 can participate in the research of regulating osteogenic differentiation. Increased expression can promote bone repair and osteogenic differentiation.^25^, ^26^, ^27^ Similarly, previous studies have confirmed that high‐fat diet can reduce the expression of Hoxa10 in mice and inhibit bone development.^28^ In this study, we confirmed that the high‐fat environment can affect the expression of Hoxa10, and we found that its expression can affect the osteogenic differentiation and fracture healing of BMSCs. We also found that H19 and Hoxa10 have the same expression trend, so we next consider whether the competing endogenous RNA (ceRNA) mechanism is involved in the process of obesity affecting fracture healing. Therefore, we use bioinformatics methods to screen the intermediate miRNAs affected by these genes.

In this study, we found that miR‐467 may be involved in the regulation of H19 and Hoxa10. We verified the targeting relationship between the three through the dual luciferase report assay and found that the regulatory relationship between the three can affect the osteogenic differentiation of BMSCs through ALP and ARS staining and osteogenic gene detection. At present, there are few studies on miR‐467, and previous studies have confirmed its correlation with the regulation of angiogenesis.^29^, ^30^ In this study, we first discovered the role of miR‐467 in osteogenic differentiation and fracture healing. This effect may be achieved by regulating the expression of Hoxa10.

In summary, this study further found that H19 plays an important role in the poor healing of fractures caused by obesity by combining with previous studies. Through further its source, we found that it affects further osteogenic differentiation or fracture healing through BMSCs‐derived exosomes. It is worth noting that the specific surface ligands of exosomes ensure that they bind to target cells and deliver their contents, ultimately regulating specific biological functions. It has been confirmed that exosomal transplantation and direct stem cell transplantation have similar therapeutic effects and functional characteristics.^31^ Although this therapy provides a feasible direction for future treatment, its ability to target targeted and the feasibility of clinical application still need more data support. Our study found that the application of BMSCs‐derived exosomes from normal sources can inhibit the phenomenon of poor fracture healing caused by high‐fat diet. The specific mechanism of this phenomenon may be caused by the regulation of Hoxa10 expression by lncRNA H19 competitively binding miR‐467. In this study, we verified the targeting relationship and regulatory functions of these three and provided new ideas and theoretical basis for future exosomes to treat poor fracture healing.

## CONFLICT OF INTEREST

The authors confirm that there are no conflicts of interest.

## AUTHOR CONTRIBUTION

**Yijun Wang:** Data curation (equal); Investigation (equal); Methodology (equal); Supervision (equal); Writing‐review & editing (equal). **Wentao Chen:** Conceptualization (equal); Data curation (equal); Project administration (equal). **Liang Zhao:** Formal analysis (equal); Validation (equal); Visualization (equal); Writing‐original draft (equal). **Yadong Li:** Conceptualization (equal); Methodology (equal); Software (equal). **Zhenyu Liu:** Data curation (equal); Visualization (equal); Writing‐original draft (equal). **Hua Gao:** Investigation (equal); Methodology (equal). **Xiaodong Bai:** Conceptualization (equal); Methodology (equal); Resources (equal). **Baojun Wang:** Conceptualization (equal); Data curation (equal); Software (equal); Visualization (equal); Writing‐original draft (equal).

## ETHICAL APPROVAL

All the patients involved in this study received informed consent. All animal experimental procedures were conducted in accordance with regulations from ethics committee in Beijing Friendship Hospital.

## INFORMED CONSENT

Informed consent was obtained from all individual participants included in this study.

## Supporting information

Figure S1

Figure S2

Figure S3

Figure S4

Table S1

Table S2

Supplementary Material

## Data Availability

The data set supporting the conclusions of this article is available in the corresponding author repository.
