# Supplementary figures and images for: Obesity regulates miR‐467/HoxA10 axis on osteogenic differentiation and fracture healing by BMSC‐derived exosome LncRNA H19
Source: J Cell Mol Med. 2021 Jan 20;25(3):1712–24. doi: 10.1111/jcmm.16273 (PMC7875915; doi:10.1111/jcmm.16273)

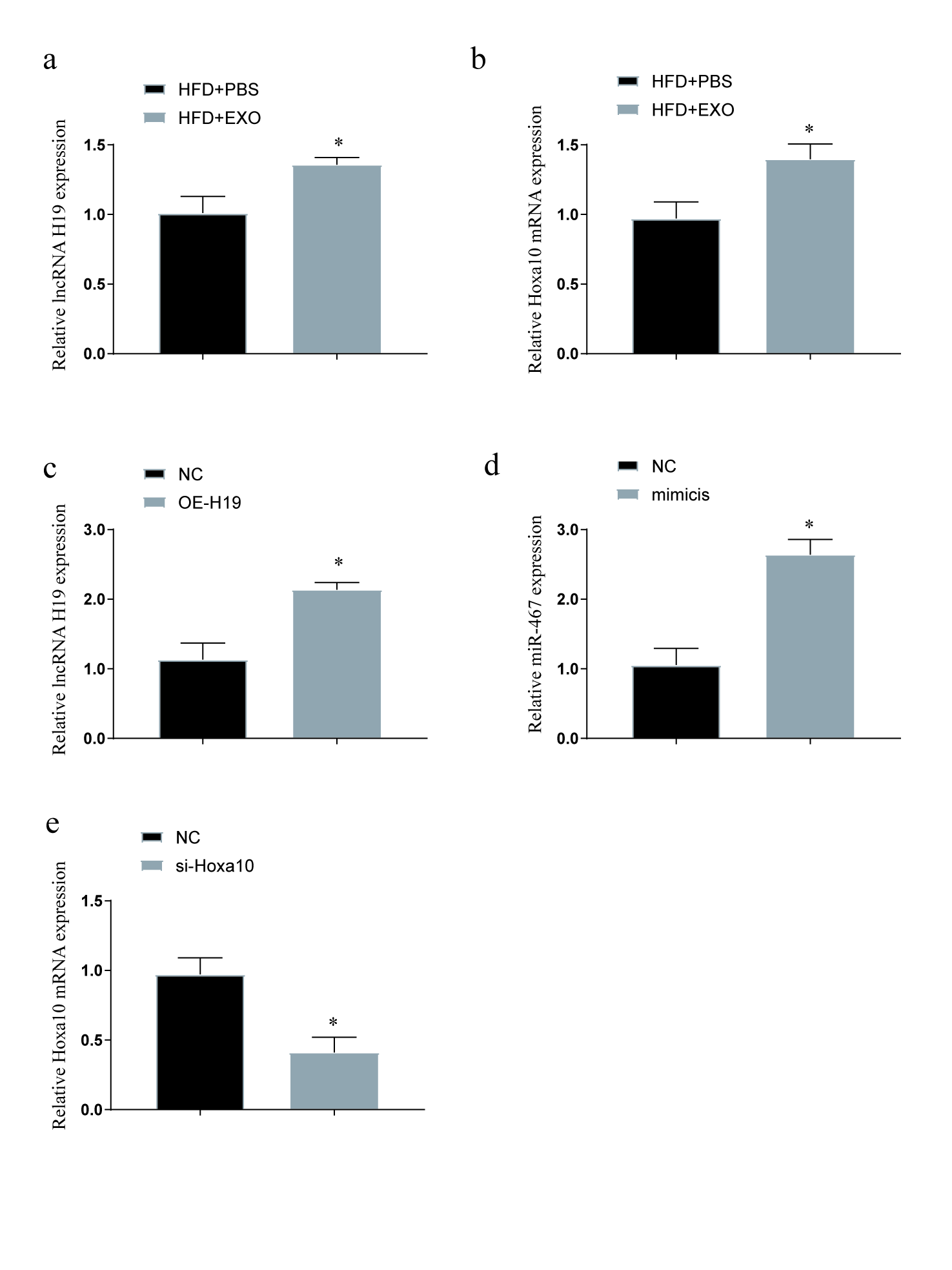

Supplement: Supplementary file 1 — Figure S1 [file JCMM-25-1712-s001.tif]

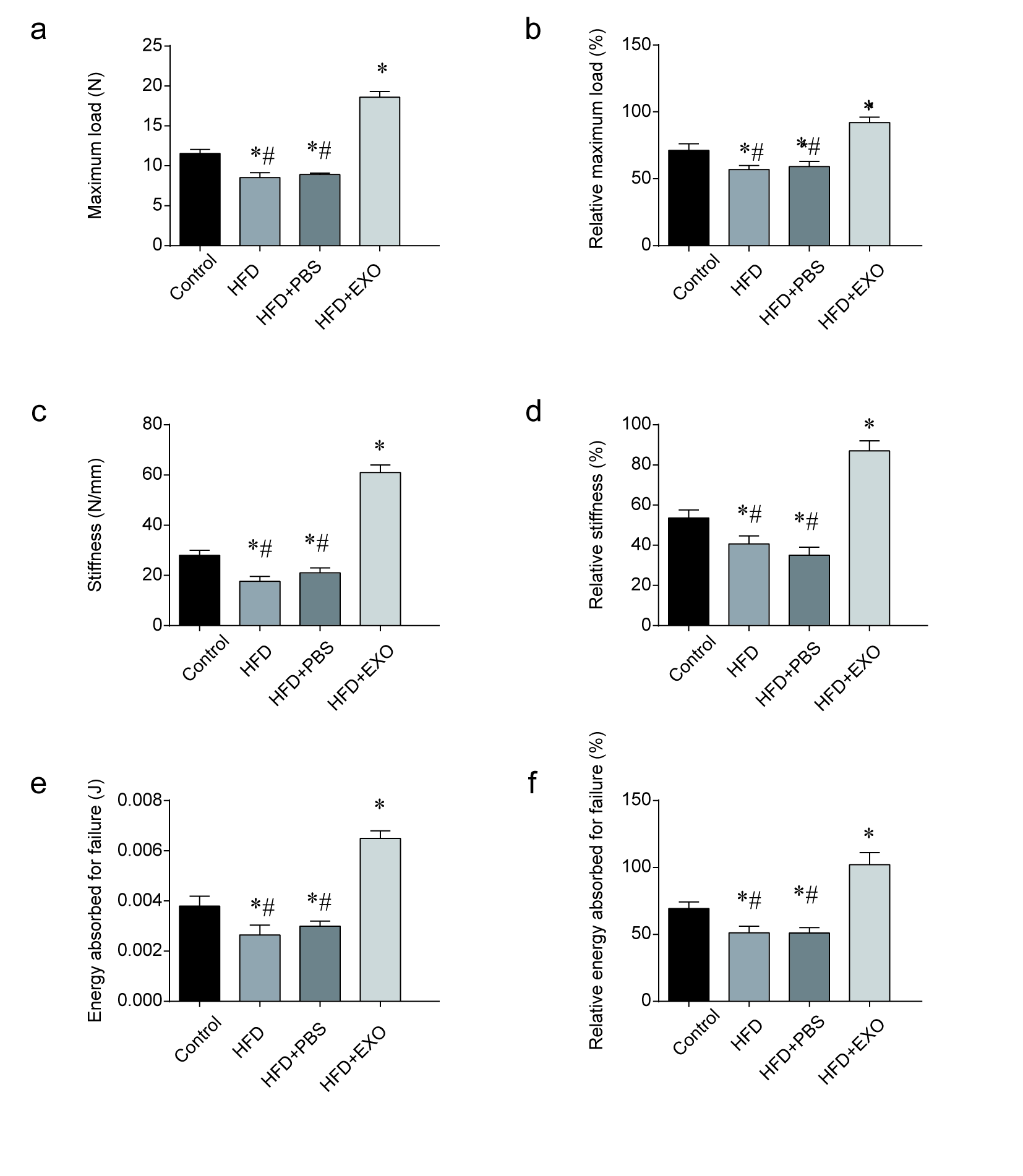

Supplement: Supplementary file 2 — Figure S2 [file JCMM-25-1712-s002.tif]

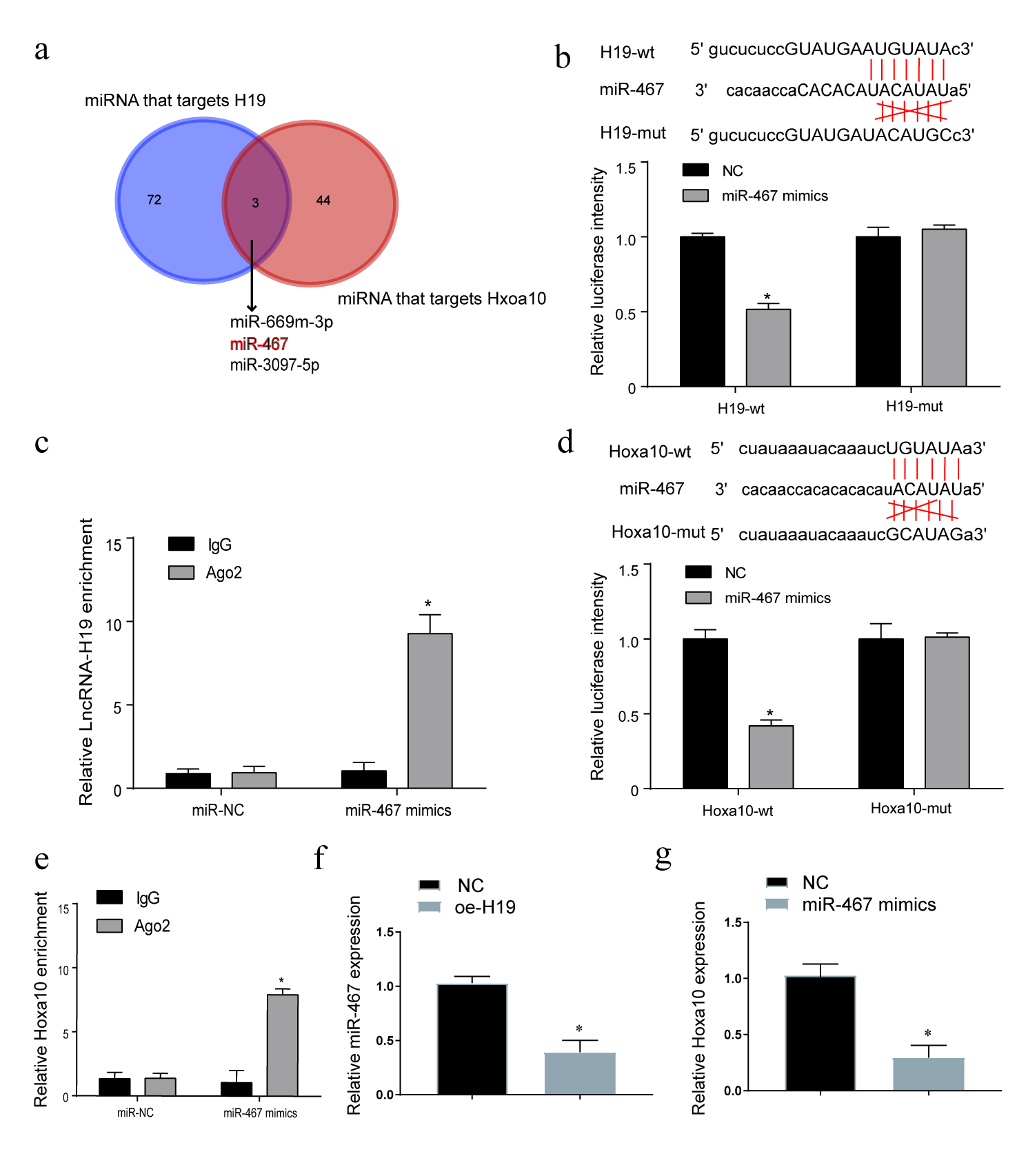

Supplement: Supplementary file 3 — Figure S3 [file JCMM-25-1712-s003.tif]

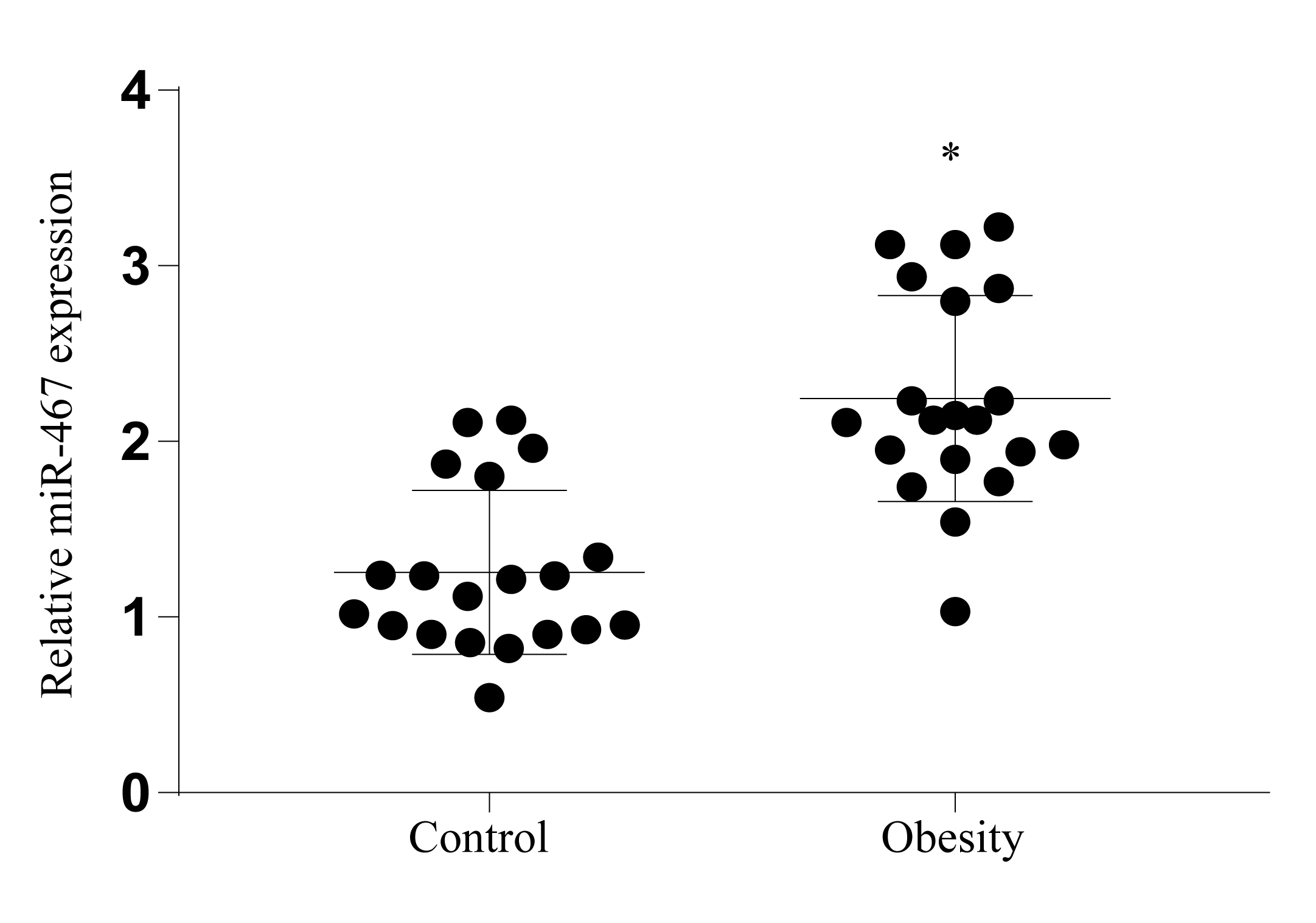

Supplement: Supplementary file 4 — Figure S4 [file JCMM-25-1712-s004.tif]
