# Supplementary material for: Obesity regulates miR‐467/HoxA10 axis on osteogenic differentiation and fracture healing by BMSC‐derived exosome LncRNA H19
Source: J Cell Mol Med. 2021 Jan 20;25(3):1712–24. doi: 10.1111/jcmm.16273 (PMC7875915; doi:10.1111/jcmm.16273)
Supplement: Supplementary file 5 — Table S1 [file JCMM-25-1712-s005.docx]

**Table S1. Clinical characteristics of obese and non-obese subjects enrolled in the study.**

|  | Obese fracture patients | Healthy fracture patients |
| --- | --- | --- |
| N | 20 | 20 |
| Gender (F/M) | 9/11 | 10/10 |
| Age | 47.6±13.5 | 51.4±10.1 |
| BMI (Kg/m^2^) | 43.96±4.03 | 19.6±0.6 |
| Hypertension | 0 | 0 |
| Diabetes | 0 | 0 |
