# Supplementary material for: Obesity regulates miR‐467/HoxA10 axis on osteogenic differentiation and fracture healing by BMSC‐derived exosome LncRNA H19
Source: J Cell Mol Med. 2021 Jan 20;25(3):1712–24. doi: 10.1111/jcmm.16273 (PMC7875915; doi:10.1111/jcmm.16273)
Supplement: Supplementary file 6 — Table S2 [file JCMM-25-1712-s006.docx]

**Table S2. The primers sequences used for qRT-PCR**

| PCR primers sequences | Forward (5'-3') | Reverse (5'-3') |
| --- | --- | --- |
| miR-467 | GCGCGATATACATACACACACCAACAC | AACGCTTCACGAATTTGCGT |
| H19 | AGACCTGGGCAGTGAAGGTA | TATGTGCCATTCTGCTGCGA′ |
| U6 | CTCGCTTCGGCAGCACACA | AACGCTTCACGAATTTGCGT |
| Hoxa10 | TTCGCCGGAGAAGGACTC | TCTTTGCTGTGAGCCAGTTG |
| RUNX2 | GAACCAAGAAGGCACAGACAGA | GGCGGGACACCTACTCTCATAC |
| OPN | TGAATGGTGCATACAAGGCCATCC | TTCATAACTGTCCTTCCCACGGCT |
| OCN | GCTTCCTGGAACAGCAAAAC | GCTTCCTGGAACAGCAAAAC |
| GAPDH | GAAGGTGAAGGTCGGAGTC | GAAGGTGAAGGTCGGAGTC |
