# Supplementary material for: Obesity regulates miR‐467/HoxA10 axis on osteogenic differentiation and fracture healing by BMSC‐derived exosome LncRNA H19
Source: J Cell Mol Med. 2021 Jan 20;25(3):1712–24. doi: 10.1111/jcmm.16273 (PMC7875915; doi:10.1111/jcmm.16273)
Supplement: Supplementary file 7 — Supplementary Material [file JCMM-25-1712-s007.docx]

**Supplementary Figure 1** **The expression of LncRNA H19 and Hoxa10 in each treatment group.**

(a-b) The expression of LncRNA H19 and Hoxa10 in each treatment group, *vs HFD-PBS group P<0.05; (c-e) The expression of the target gene in each overexpression treatment group, *vs NC group *P*<0.05. All data were means ± SD.

**Supplementary Figure 2 Mechanical testing of the callus.**

Maximal loading failure (a), stiffness (c) and energy absorbed for failure (e) are measured to reveal the mechanical property of the fracture callus at week 6 post-fracture. The relative mechanical properties to collateral side are shown in (b) (Relative maximum load), (d) (Relative stiffness), and (f) (Relative energy absorbed for failure), respectively, *vs control *P*<0.05, #vs HFD+EXO group *P*<0.05. All data were means ± SD.

**Supplementary Figure 3 Prediction and verification of target genes of LncRNA H19 and Hoxa10.**

(a) Venn diagram screens the common target miRNA situation of lncRNA H19 and Hoxa10; (b-c) Dual luciferase report and RIP assay verifies the targeting of LncRNA H19 and miR-467, *vs NC group P<0.05; (d-e) Dual luciferase report and RIP assay verifies the targeting of miR-467 and Hoxa10, *vs NC group P<0.05 (f) The effect of overexpression of H19 on the expression of miR-467, *vs NC group P<0.05; (g) The effect of overexpression of miR-467 on the expression of Hoxa10, *vs NC group P<0.05. All data were means ± SD.

**Supplementary Figure 4 The expression of miR-467 in clinical sample**

Expression of lncRNA H19 and Hoxa10 in clinical samples, *vs control *P*<0.05. All data were means ± SD.
